# Supplementary material for: Transport to the Slaughterhouse Affects the Salmonella Shedding and Modifies the Fecal Microbiota of Finishing Pigs
Source: Animals (Basel). 2020 Apr 13;10(4):676. doi: 10.3390/ani10040676 (PMC7222783; doi:10.3390/ani10040676)
Supplement: Supplementary file 1 [file animals-10-00676-s001.zip › Figure_S2.pdf]

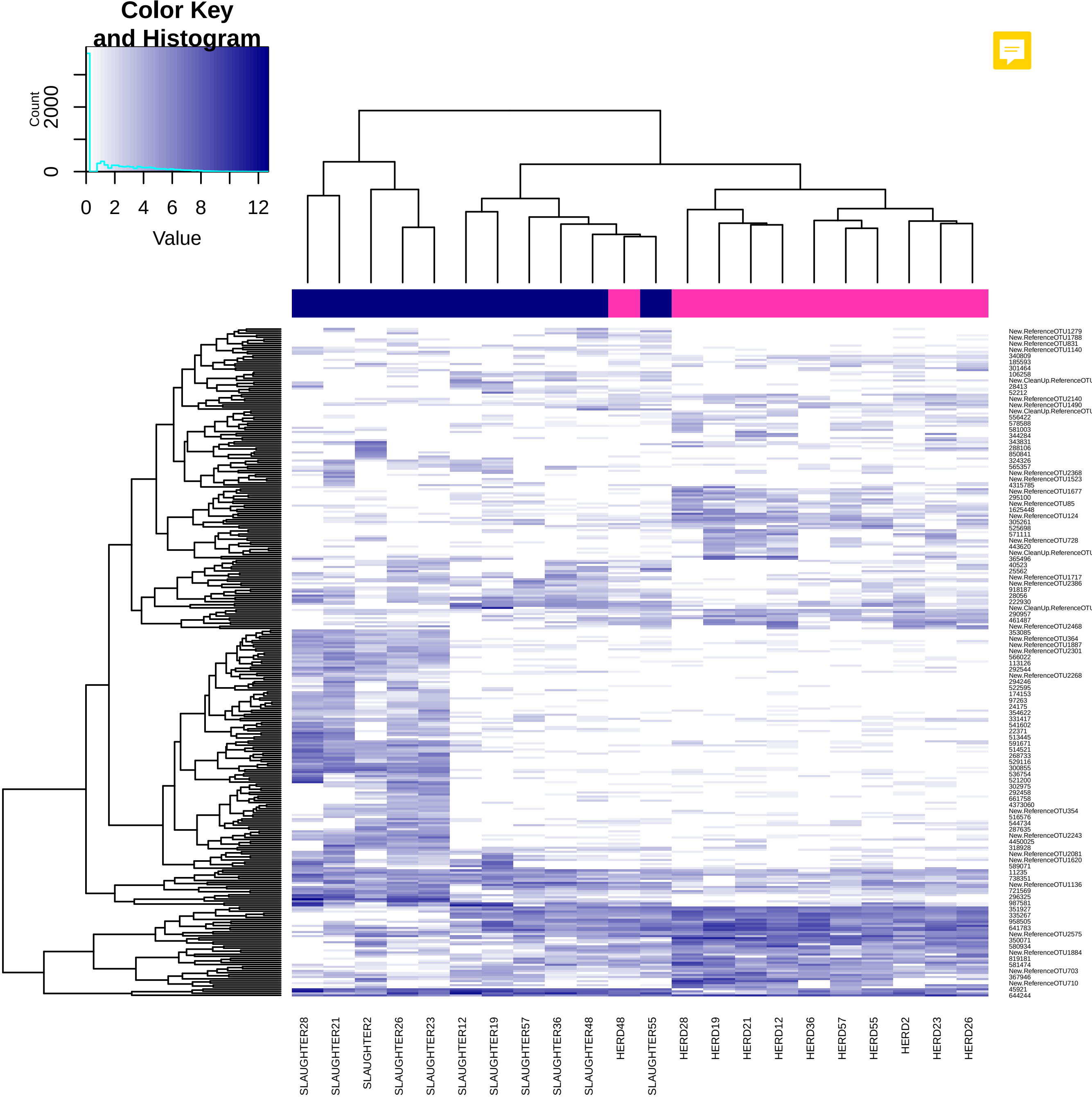

Color Key
and Histogram
Count
0
2000
0
2
4
6
8
12
Value
New.ReferenceOTU1279
New.ReferenceOTU1788
New.ReferenceOTU831
New.ReferenceOTU1140
340809
185593
301464
106258
New.CleanUp.ReferenceOTU
28413
52212
New.ReferenceOTU2140
New.ReferenceOTU1490
New.CleanUp.ReferenceOTU
556422
578588
581003
344284
343831
288106
850841
324326
565357
New.ReferenceOTU2368
New.ReferenceOTU1523
4315785
New.ReferenceOTU1677
295100
New.ReferenceOTU85
1625448
New.ReferenceOTU124
305261
525698
571111
New.ReferenceOTU728
443620
New.CleanUp.ReferenceOTU
365496
40523
25562
New.ReferenceOTU1717
New.ReferenceOTU2386
918187
28056
222930
New.CleanUp.ReferenceOTU
290957
461487
New.ReferenceOTU2468
353085
New.ReferenceOTU364
New.ReferenceOTU1887
New.ReferenceOTU2301
566022
113126
292544
New.ReferenceOTU2268
294246
522595
174153
97263
24175
354622
331417
541602
22371
513445
591671
514521
268733
529116
300855
536754
521200
302975
292458
661758
4373060
New.ReferenceOTU354
516576
544734
287635
New.ReferenceOTU2243
4450025
318928
New.ReferenceOTU2081
New.ReferenceOTU1620
589071
11235
738351
New.ReferenceOTU1136
721569
296325
987581
351927
335267
958505
641783
New.ReferenceOTU2575
350071
580934
New.ReferenceOTU1884
819181
581474
New.ReferenceOTU703
367946
New.ReferenceOTU710
45921
644244
SLAUGHTER28
SLAUGHTER21
SLAUGHTER2
SLAUGHTER26
SLAUGHTER23
SLAUGHTER12
SLAUGHTER19
SLAUGHTER57
SLAUGHTER36
SLAUGHTER48
HERD48
SLAUGHTER55
HERD28
HERD19
HERD21
HERD12
HERD36
HERD57
HERD55
HERD2
HERD23
HERD26
