# Supplementary figures and images for: Transport to the Slaughterhouse Affects the Salmonella Shedding and Modifies the Fecal Microbiota of Finishing Pigs
Source: Animals (Basel). 2020 Apr 13;10(4):676. doi: 10.3390/ani10040676 (PMC7222783; doi:10.3390/ani10040676)

**Alpha diversity**

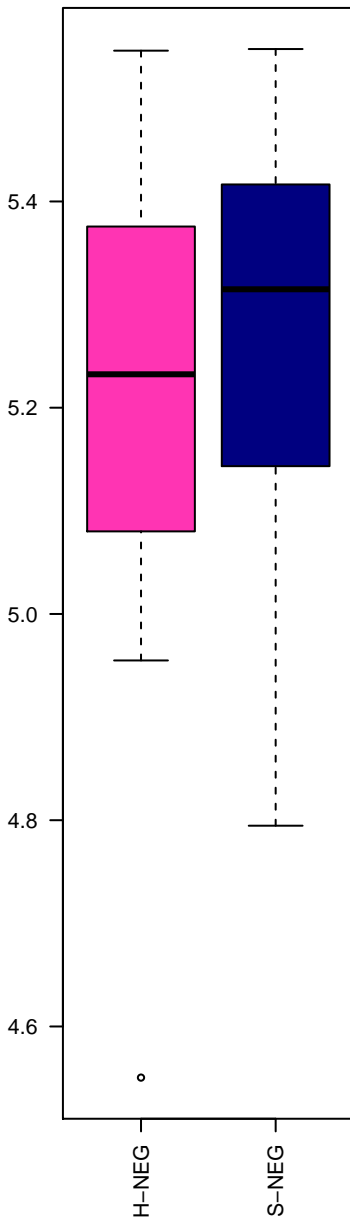

**Beta diversity**

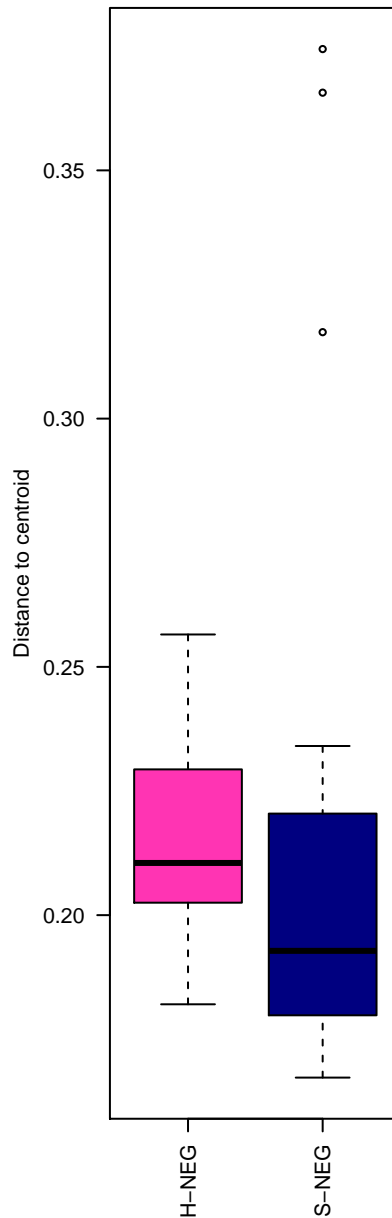

**Richness**

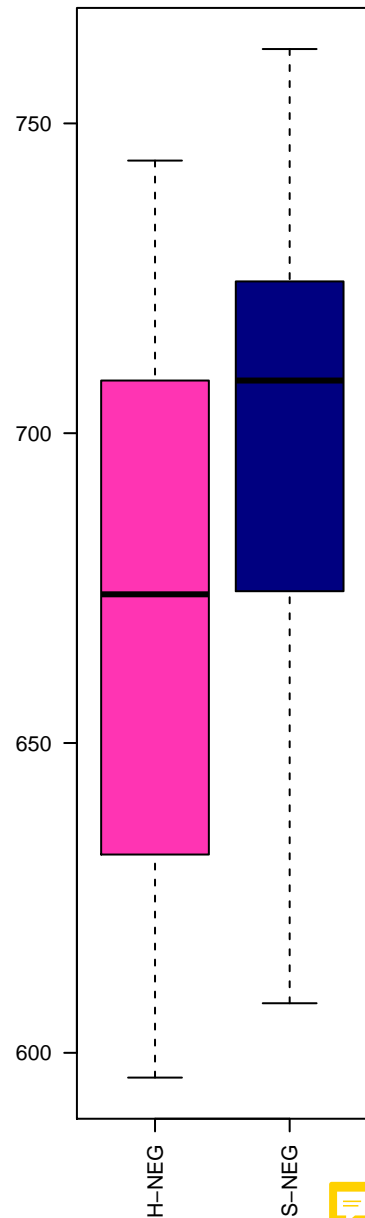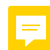

Supplement: Supplementary file 1 [file animals-10-00676-s001.zip › Figure_S1.pdf]
